# Annexin-Phospholipid Interactions. Functional Implications

**DOI:** 10.3390/ijms14022652

**Published:** 2013-01-28

**Authors:** María Antonia Lizarbe, Juan I. Barrasa, Nieves Olmo, Francisco Gavilanes, Javier Turnay

**Affiliations:** Department of Biochemistry and Molecular Biology I, Faculty of Chemistry, Complutense University, Madrid 28040, Spain; E-Mails: lizarbe@bbm1.ucm.es (M.A.L.); jbarrasa@bbm1.ucm.es (J.I.B.); nieves@bbm1.ucm.es (N.O.); pacog@bbm1.ucm.es (F.G.)

**Keywords:** annexin, calcium binding, membrane aggregation, membrane binding, phospholipid binding, phosphatidylserine

## Abstract

Annexins constitute an evolutionary conserved multigene protein superfamily characterized by their ability to interact with biological membranes in a calcium dependent manner. They are expressed by all living organisms with the exception of certain unicellular organisms. The vertebrate annexin core is composed of four (eight in annexin A6) homologous domains of around 70 amino acids, with the overall shape of a slightly bent ring surrounding a central hydrophilic pore. Calcium- and phospholipid-binding sites are located on the convex side while the *N*-terminus links domains I and IV on the concave side. The *N*-terminus region shows great variability in length and amino acid sequence and it greatly influences protein stability and specific functions of annexins. These proteins interact mainly with acidic phospholipids, such as phosphatidylserine, but differences are found regarding their affinity for lipids and calcium requirements for the interaction. Annexins are involved in a wide range of intra- and extracellular biological processes *in vitro*, most of them directly related with the conserved ability to bind to phospholipid bilayers: membrane trafficking, membrane-cytoskeleton anchorage, ion channel activity and regulation, as well as antiinflammatory and anticoagulant activities. However, the *in vivo* physiological functions of annexins are just beginning to be established.

## 1. Introduction

Annexins are a widely distributed multigene superfamily of structurally related calcium-dependent membrane-binding proteins that show a characteristic tetrad structure of homologous internal repeats. They are expressed in many organisms from protists to higher eukaryotes, including plants [1–4]. In 1999, the nomenclature system for these proteins was accorded based on the molecular phylogeny and comparative genomic analysis [5]. They are now named “annexin” followed by a capital letter: A for human annexins and their cognate orthologs; B for other animal annexins, mainly invertebrate; C for *Mycetozoa* and fungi; D for plants; and E for *Protista*. Annexins have diverged significantly, despite their gross structural similarity, in terms of their gene regulation, tissue-specific expression patterns, subcellular localization of different isoforms, and features peculiar to individual subfamilies. Annexins are involved in several cellular functions, like membrane trafficking, exocytosis, endocytosis, membrane-cytoskeleton interactions, regulation of membrane protein activities, calcium channel activity and signal transduction, among others. Moreover, although annexins are mainly cytosolic, they can also be found as extracellular proteins exerting additional functions as anticoagulant and antiinflamatory proteins, or mediating the interaction with other extracellular proteins [1,2]. In this review, we have focused on the structure and the interaction of annexins, mainly from the A subfamily, with phospholipid membranes, and the implication of these interactions on their cellular functions.

## 2. Annexin Structure

### 2.1. The Annexin Core

**Figure 1:**
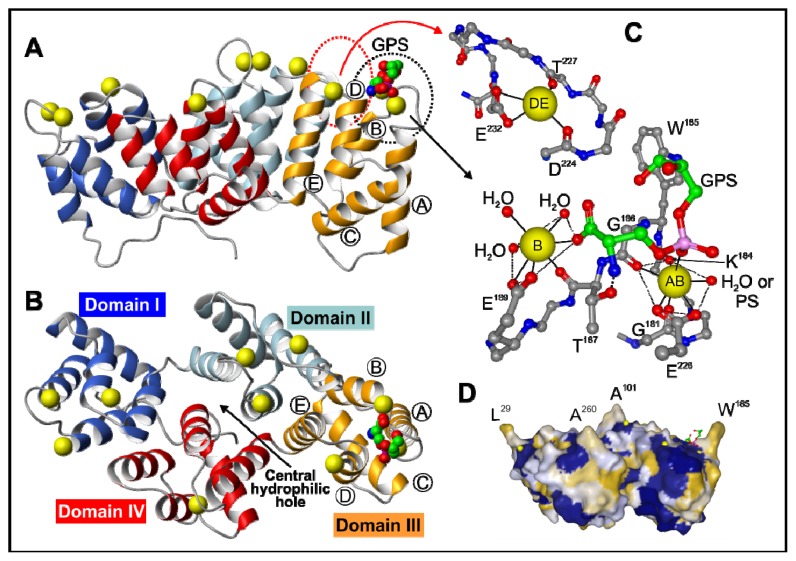
Three-dimensional structure of rat annexin A5. The three-dimensional structure of rat annexin A5 with the four Type II calcium-binding sites saturated with calcium was obtained using MOLMOL [11] and is based on the protein data bank (PDB) file 1A8A [12]. A lateral view (**A**), a top view of the molecule (**B**), a detail of the interactions in Domain III with calcium and the polar head of phosphatidylserine (PS) (**C**), and a hydrophobicity surface representation (**D**) are shown. The four domains in the protein core are represented in different colors: I, blue; II, cyan; III, orange; and IV, red. The letters assigned to the α-helices are shown only in Domain III. Calcium ions are represented by yellow spheres. Panel B allows a clear view of the central hydrophilic hole; the different domains are indicated. The calcium-dependent binding of glycerophosphoserine (GPS) to Domain III of the annexin core is shown in (**C**) together with the coordinations that bind calcium to the protein at the “AB”, “B” and “DE” sites. The carbon atoms of the polypeptide backbone and the lateral chains of key residues for the interaction with calcium are represented in grey, whereas carbons in GPS are green (oxygen, red; nitrogen, blue; phosphorus, magenta).

All the members of this family of proteins are structurally characterized by the presence of a highly conserved core composed of four (eight in annexin A6) homologous domains of about 70 amino acids showing a similar three-dimensional structure highly conserved throughout annexin evolution [6]. Inside each domain, a sequence of 17 amino acid residues is found which presents an even higher degree of conservation. Annexin A5 was the first member of this family to be crystallized and its structure to be studied by X-ray diffraction [6,7]. Since then, several other annexins have been crystallized, and all of them present an almost identical three-dimensional arrangement in the protein core. The overall shape of these molecules is that of a slightly bent disc (Figure 1A) where the four repeated domains are arranged around a central hydrophilic hole (Figure 1B); this hole could be responsible for the voltage-dependent calcium channel activity reported for several annexins when bound to membranes (A1, A2, A5–A7, B12) [8–10]. Each domain contains a four α-helix bundle (helices A, B, D & E) organized in a cylindrical way and capped by a fifth α-helix (C) that is located on the concave surface of the annexin molecule (Figure 1A; helices are labeled in Domain III). Calcium ions bind on the convex side of the molecule. There are up to three possible coordination sites per domain, the so-called Type II or “AB” site being the one that shows higher affinity for calcium. In this location, calcium binds to carbonyl oxygens in the loop connecting the A and B helices and to a bidentate carboxyl group from a glutamic or aspartic acid residue located around 40 residues downstream in the loop connecting helices D and E (Figure 1).

### 2.2. The N-Terminal Domain

In contrast to the annexin core, which shows a clear homology, sequence analysis of annexins shows the great diversity acquired by their *N*-terminal extension during evolution. These domains are variable both in length and amino acid sequence. In human annexins, the *N*-terminus region ranges from a few residues to 200 or more amino acids and, based on the length of this region, they can be classified into three groups. The first one includes those members with a short extension below 20–21 amino acids, such as annexins A3, A4, A5, A6, A8, A10 and A13a. A second group includes annexins with an intermediate *N*-terminus, up to 55 residues, such as annexins A1, A2, A9 and A13b. Finally, a third group can be established with annexins that possess a long *N*-terminus with more than 100 amino acid residues (annexins A7 and A11).

Even though the *N*-terminus of annexins is significantly smaller than the *C*-terminal protein core, it is essentially involved in the structural and functional peculiarities of each member of this superfamily of calcium-binding proteins [13,14]. The *N*-terminal extension contributes significantly to the stability of the overall structure; it is also essential for self-association and mediates the interaction with other proteins, mainly from the S100 family (also calcium binding proteins but with the EF-hand motif). Moreover, post-translational modifications of this region (phosphorylation, acetylation, myristoylation, proteolysis, *etc.*) modify the structure of key regions of the protein core, even if they are located at the opposite side of the molecule. Consequently, the *N*-terminal domain of these proteins has to be considered as the main regulatory element of both annexin structure and function.

Crystallographic data have revealed that the *N*-terminal domain determines the structural arrangement of protein regions located on the opposite concave region of the annexin molecule binding together Domains I and IV [6,12,15–18], at least in annexins with a short *N*-terminal domain such as annexin A5. Interestingly, using circular dichroism and fluorescence spectroscopy, we have found that an annexin A5 mutant with a truncated *N*-terminus also presents a more solvent-exposed IIIAB loop [19]. Thus, it seems that the interaction of the *N*-terminus with Domain IV in the concave side is transmitted to the loop located in Domain III at the opposite side of the molecule, as the truncation of the *N*-terminus forces a conformational rearrangement of the loop. Moreover, phosphorylation of Thr6 in annexin A4, that involves the displacement of the *N*-terminus, also impairs its ability to induce vesicle aggregation [20]. A similar effect is observed in annexin A1 after phosphorylation of Ser27; however, it does not affect binding to membranes and lateral self-association [21].

**Figure 2:**
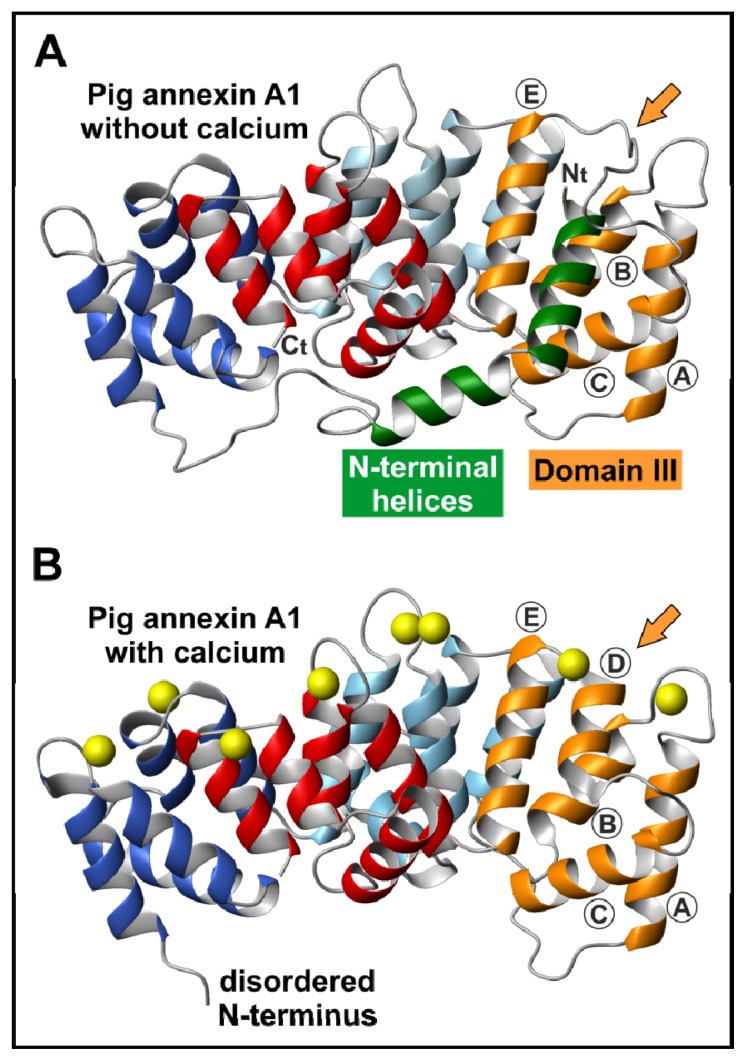
Crystal structure of full-length pig annexin A1 in the absence and presence of calcium. The three-dimensional structure of pig annexin A1 in the absence of calcium (**A**) (PDB file 1HM6) [22] shows the intact *N*-terminus with the appearance of two consecutive α-helices (residues 2–18 and 19–28; green), the most *N*-terminal one being inserted into Domain III (orange) with the consequent disappearance of Helix 3D. The structure of this annexin in the presence of calcium (**B**) (PDB file 1MCX) [24] does not show the disordered *N*-terminus; as observed, Helix 3D is refolded when the *N*-terminal helix moves out of the protein core. Arrows show the most affected region of the protein core after the interaction with calcium. Three-dimensional structures were drawn using MOLMOL.

The crystal structure of an intermediate annexin with its complete *N*-terminus in the absence of calcium was first determined for annexin A1 [22]. In this protein, as expected from sequence homology to other crystallized annexins and from the structure of the truncated form, the *N*-terminus runs first through the concave side of the molecule from Domain I to Domain IV as an extended coil. This region is constituted by amino acids 28 to 41, which are preceded by two consecutive amphipathic α-helices (Figure 2). The second helix includes amino acids 18 to 27 and interacts with Domain IV. It is directed towards Domain III where it turns in position 18. The most *N*-terminal helix (residues 2–17) is inserted into Domain III and displaces Helix IIID, which in turn becomes an extended loop over the convex side of the molecule. It has been shown that the interaction of annexin A1 with phosphatidylserine (PS) bilayers increases the *N*-terminal degradation susceptibility and the accessibility of disulfide bridges [23]. Calcium binding to annexin A1 induces a conformational rearrangement mainly consisting of the refolding of Helix IIID and the release of the *N*-terminal α-helix from the protein core, as deduced from the crystal structure of the entire protein in the presence of calcium [24]. The release of the *N*-terminus allows the binding of S100A11 in a calcium-dependent fashion. In this sense, the *N*-terminus would act as a regulatory region that modulates the possible interactions of the protein core with either membranes or other proteins.

In annexins with a long or intermediate *N*-terminus, some α-helical stretches can be predicted. These α-helical sub-domains could be involved in the interaction with other proteins from the S100 family, these associations being essential for their function. In annexin A11, with a long unordered *N*-terminus, residues 45–62 are predicted to form an amphipathic α-helix, and it is tempting to suggest a similar interaction between this helix and the protein core through Domain III. However, we have shown by fluorescence acrylamide quenching that the unique tryptophan residue in Position 23 is completely exposed to the solvent suggesting that this region does not interact with the protein core [25].

## 3. Annexin Binding to Phospholipid Membranes

### 3.1. Calcium-Dependent Phospholipid Binding

As previously mentioned, annexins are mainly characterized by their ability to reversibly interact with membranes in a calcium-dependent manner. Although this is true for the vast majority of the members of this family of proteins, some members, such as mammalian annexin A9, do not bind calcium but still are able to interact with biological membranes through different mechanisms. Human annexin A5 was the first annexin crystallized in 1990 [6,26]. From then on, numerous crystal structures have been reported and are available through the Protein Data Bank. These structures have allowed an extensive analysis of the calcium binding sites in annexins. Figure 1 shows the structure of rat annexin A5 saturated with calcium (PDB 1A8A); ten calcium ions are coordinated with specific residues in the convex membrane-binding surface of the protein. In fact, calcium-binding measurements with annexins in solution indicate that these proteins may bind up to 10–12 Ca^2+^ ions, although with different affinities [27–29]. Differences in the stoichiometry of the binding have been associated with the ability of annexins to establish trimers upon membrane binding or not: Trimer-forming annexins A5 and B12 bind around 12 mol of Ca^2+^/mol of protein whereas nontrimer-forming annexins A1 and A2 show a lower stoichiometry (3–4 mol of Ca^2+^/mol of protein). In addition, the A5/B12 group of annexins binds to bilayers in the liquid-crystal phase but not in the gel phase whereas the opposite occurs in the A1/A2 group, suggesting that there is a complementarity between the spacing of the Ca^2+^-binding sites and the spacing of the phospholipid head groups in the bilayers [28]. In any case, most annexins contain two to four Type II (“AB”) high-affinity Ca^2+^-binding sites located in the loop between Helices A and B. In addition, annexins may contain two additional calcium binding sites with lower affinity, named Type III, located at the *N*-terminus of Helix B (“B” site) or in the loop between Helices D and E (“DE” site) (Figure 1). Mutagenesis studies indicate that the “AB” sites are required for attachment to membranes, while the B or DE sites are not sufficient for membrane binding, although their presence increases the binding affinity [30,31].

Each of the calcium-binding sites shows a structural pattern in which the Ca^2+^ ion is coordinated by seven oxygen atoms forming a more or less regular pentagonal bipyramid. Five oxygens lie in the equatorial plane and two in axial positions. In the main “AB” calcium-binding sites, five oxygens belong to the protein: A bidentate carboxylate group from an aspartic or glutamic acid side chain located 40 residues downstream of a conserved glycine present in the interhelical AB loop (equatorial plane) and three oxygens from carbonyl groups form peptides bonds within the loop. The remaining two oxygens belong to water molecules in the absence of phospholipids, or to phosphate groups from the polar heads of two phospholipid head groups.

Figure 1C shows a detail of the “AB” calcium-binding site in Domain III of rat annexin A5 indicating the residues involved in coordination as well as one glycerophosphoserine (GPS) moiety that resembles the head group of PS, the main lipidic ligand of annexins in biological membranes. The “B” site is close to the “AB” site and collaborates in PS binding, as one of the oxygen atoms of the carboxyl group of serine from PS is coordinated with the calcium ion at this position. The annexin AB loop conformation is almost identical to that observed in crystals saturated only with calcium or with calcium and bound GPS. No other phospholipid, besides PS, has oxygens that may coordinate to the calcium at the “B” site. Moreover, the serine α-amino group appears to stabilize the interaction of the phosphoserine head by establishing a hydrogen bond with the hydroxyl group of a conserved threonine residue (T^187^ in Domain III of rat annexin A5; Figure 1C). All these stabilizing interactions can explain why PS is the “preferred” phospholipid ligand of annexins, as this calcium binding seems to be specifically designed to fit the serine head group. In fact, the crystal structure of this annexin with bound glycerophosphoethanolamine (resembling phosphatidylethanolamine (PE); PDB file 1A8B) shows that this head group is only coordinated with the “AB” site, but no additional stabilizing interactions occur [12].

The GPS molecule shown in the crystal structure of rat annexin A5 (Figure 1C) represents the so-called “apical” PS molecule that binds to the “AB” site with high affinity [32], and that is present in the four domains of almost all vertebrate annexins. In addition, the calcium ion bound to this site may coordinate with the phosphate group of an additional PS molecule in the “equatorial” binding site. This additional site involves not only the coordination with calcium, but also six or seven protein ligands from residues at the *C*-terminus of Helix A, the CD loop, and Helix D. This consensus sequence is found with slight variations in all vertebrate annexins, but only in Domains I and II, not being present in Domains III and IV [32]. The existence of this “equatorial” PS binding site was determined by molecular modeling, as radiocrystallography or nuclear magnetic resonance (NMR) hardly provide essential information at the atomic resolution when a phospholipid bilayer is concerned. Mutation of this consensus sequence in annexin A5 did not suppress calcium or PS binding to the “AB” site, but required more calcium to compensate for the loss of PS binding in the equatorial site.

The affinity of the “B” site for calcium is quite low as only three oxygens from the protein contribute to calcium coordination: A bidentate carboxyl group and a carbonyl group from a peptide bond (Figure 1C). In the absence of PS, four positions are occupied by oxygens from water molecules, but when PS is present, one of the water molecules is replaced by a carboxyl oxygen from the phosphoserine head group. The establishment of tertiary annexin-Ca^2+^-PS complexes strongly increases the affinity of annexins towards calcium possibly due to the replacement of solvent molecules by oxygens from the phospholipid polar head. In fact, crystallographic data as well as solution measurements are consistent with an increase in affinity of two or three orders of magnitude [33–35].

A third possible phospholipid head group may bind to each annexin domain via the “DE” calcium-binding site. This additional calcium ion is coordinated with four oxygens from the loop between Helices D and E: A bidentate carboxyl group from the lateral chain of an aspartic or glutamic acid at the beginning of Helix E, and two carbonyl oxygens from peptide bonds within the loop. The remaining three coordinations are occupied by water in the absence of phospholipids (Figure 1C). In the presence of phospholipids, two water molecules are replaced by oxygens from the polar head group, as suggested by molecular dynamics simulation [36].

All in all, up to three phospholipid polar head groups can interact with each domain of the annexin core structure. Additional stabilizing interactions can be established between the convex surface of the annexin molecules and phospholipid bilayers. Figure 1D shows the molecular surface of rat annexin A5 indicating the hydrophobic (yellow) and hydrophilic (blue) regions. When calcium is bound to the “AB” sites, hydrophobic lateral chains are exposed in the otherwise mainly hydrophilic convex surface. These hydrophobic residues are highly conserved in the AB loops and may contribute to the interaction with bilayers by establishing van der Waals forces with the hydrophobic acyl chains of phospholipids. However, these residues do not penetrate deeply into the bilayer as observed from the behavior of the fluorescence spectra of the tryptophan residue located in the AB loop of Domain III of several vertebrate annexins [35,37].

Annexins are mainly cytosolic proteins that bind preferentially to the major phospholipidic components of the intracellular leaflet, PS and PE, although they exhibit a marked preference for PS over PE [2,38]. In addition, annexins may bind other phospholipids at physiological pH, such as the negatively charged ones phosphatidic acid (PA), phosphatidylglycerol (PG) or phosphatidylinositol (PI). Some annexins also show further, more specific interactions with membrane lipids; for example, annexins A2 and A8 bind phosphatidylinositol-4,5-bisphosphate (PIP_2_) [39–41]. In general, the net charge of the polar head of phospholipids seems to be important for recruiting annexins to the membrane. However, the molecular interaction of the lipid head group with specific protein residues strongly influences the affinity with which the lipids bind and to which site they do. This could explain why PS binds more tightly than PI to annexins even though they have the same negative charge at physiological pH, as well as why uncharged PE binds to annexins whereas phosphatidylcholine (PC) does not.

Annexins do not require all four domains from the protein core to interact with phospholipid bilayers. In fact, different annexins show preferences for one domain or another in order to establish the interactions with membranes. On this idea, mutational analysis has suggested that Domain I in annexin A5 plays a prominent role in the lipid-binding process, although the rest of the Ca^2+^ and phospholipid binding sites collaborate to stabilize the interaction. Interestingly, annexin A1 lacks the consensus Type II or “AB” calcium binding site at Domain I; moreover, this sequence is lost in all repeats of annexin A9, in Domains I and IV of annexin A2, Domains I, III and IV of annexin A10 and in Domain III of the *C*-terminal tetrad of annexin A6 [42]. In addition, the free Ca^2+^ concentrations required to initiate phospholipid binding differ markedly among different annexins and different phospholipid head groups. They range from 20 μM for the binding of annexin A5 to PS-containing liposomes, through submicromolar Ca^2+^ concentrations for the PS- and PA-binding of annexins A1 and A2, to less than 100 nM for the interaction of the annexin A2-S100A10 complex with PS-containing membranes [2,38]. This indicates that the annexin family as a whole can respond to a large spectrum of Ca^2+^ concentration changes induced by different stimuli with intracellular translocation from the cytosol to the membrane. Moreover, individual annexins are specifically designed to react only to stimuli of certain amplitude. Among vertebrate annexins, the only member that does not interact with PS-containing liposomes at submillimolar calcium concentrations is annexin A9 which lacks the Type II consensus calcium and phospholipid binding sites [43] (no data are available regarding annexin A10 that lacks three of the four binding sites). It is worth mentioning that around one third of annexin domains in the eukaryotic annexin superfamily has lost the Type II calcium binding domains. However, many of these exposed regions present a novel K/H/RGD motif (that may also appear at the loop between Domains II and III), that may have a functional role in annexin membrane interactions probably via receptor targeting [42]. Interestingly, annexin A9 (as well as annexins A1 and A2) possesses two such novel motifs in Repeats III and IV that may be responsible for the potential membrane associated physiological functions of annexin A9 [44,45].

### 3.2. Calcium-Independent Lipid Binding

Although calcium-dependent phospholipid binding is the main characteristic of the annexin superfamily of proteins, additional lipid binding mechanisms have been described in some annexins that do not require, at least directly, calcium bridging. Among others, the main parameter regulating the calcium-independent binding is the pH value. It has been shown *in vitro* that annexins A5 and A13b undergo a conformational change at mildly acidic pH (midpoints around pH 4.1 and 5.8, respectively) that resembles that observed after calcium binding (the so-called “open conformation” with exposure of the tryptophan residue located at the AB loop of Domain III) [37,46,47]. These pH values can be reached inside the cell as it is well known that pH can decrease around 1.6 units in the proximity of the membrane in regions rich in anionic phospholipids as PS [48]. The *N*-terminal half of annexin A6 (highly similar to annexin A5) also undergoes a conformational change at acidic pH, but it involves a partial denaturation of the protein core with exposure of hydrophobic regions [49]. This conformational change seems to be an intermediate state for the calcium-independent binding of annexin A5 to PS-liposomes at pH 4.0 [50], and induces leakage from PS vesicles [51]. Interaction with acidic phospholipid vesicles at slightly low pH has also been described for annexin A4, which induces leakage from these vesicles [52], and for annexin A6, suggesting that this insertion is a prerequisite for the formation of calcium channels [53]. A pH-driven calcium-independent insertion into PS-rich monolayers has also been observed in annexin A1 [54] and has been suggested for annexin A5 [55]. These observations led to postulate that annexins could penetrate into the bilayer, in contrast to the preferred location at the vesicle surface at neutral pH, possibly adopting different conformations under the two conditions.

The introduction of site directed spin labeling for the study of calcium-independent annexin-membrane interactions has allowed further advances in understanding the molecular mechanisms by which annexins interact with acidic bilayers. These studies have been carried out with annexin B12 from *Hydra* by engineering protein mutants with specific derivatized cysteines with a paramagnetic nitroxide chain. These experiments revealed that annexin B12 inserts into the lipid bilayer after undergoing a profound structural reorganization [56–60]. Electron paramagnetic resonance analysis of the loop between Helices D and E in Domain II showed that this region refolded and formed a continuous amphipathic α-helix after calcium-independent binding to membranes at mildly acidic pH. At pH 4.0, this helix assumed a transmembrane topography, while at pH around 5.0–5.5, it was peripheral and approximately parallel to the membrane; this form was reversibly converted into the transmembrane helix by lowering the pH and returned to the surface upon increasing pH [61]. These observations suggest the presence of a proton-dependent switch in annexins that harbors the information to induce membrane insertion. This insertion could explain some of the physiological properties of these proteins, such as calcium channel activity, and could also underlie its pathway of secretion.

Annexin A13 deserves a special mention regarding calcium-independent binding to membranes. This protein is the founder and most ancient member of mammalian annexins [62]. A short “a” isoform was first identified as a gut-specific annexin highly similar to annexin A5 [63]. Later on, an alternative splicing form with an insertion of 41 residues at the *N*-terminus was described: The “b” isoform [64]. Another peculiar feature of this annexin is the presence of an *N*-terminal sequence that leads to the *N*-myristoylation of the terminal glycine residue. This post-translational modification is unique among vertebrate annexins and is present in both isoforms allowing the direct interaction of these proteins with biological membranes in a calcium-independent manner. Moreover, the myristoylated form of the A13b isoform interacts better with PC and vesicles with a raft-like composition than with acidic phospholipids [37]. In addition, annexin A13 can interact with acidic phospholipid bilayers in the presence of calcium as described for other members of the annexin superfamily. Although the core of the long A13b isoform is quite similar to that of annexin A5, its requirements for calcium binding are higher (around 10-fold) probably due to the interaction of the relatively long *N*-terminus with the protein core [37]. On the other hand, a truncated form of annexin A13b lacking the first 48 amino acid residues (quite similar to the annexin A13a isoform) presented a behavior almost identical to annexin A5 regarding calcium-dependent phospholipid binding [37].

### 3.3. Annexin-Induced Vesicle Aggregation

In addition to their ability to interact with membranes, several vertebrate annexins are able to mediate vesicle aggregation, among them annexins A1, A2, A4, A6 and A7, whereas others, such as annexins A3, A5, A11 and non-myristoylated A13b, do not promote aggregation [2,25,37,65]. Interestingly, although mammalian annexin A5 lacks the ability to aggregate vesicles, we have reported that its highly similar chicken ortholog induces aggregation of vesicles containing acidic phospholipids even at low protein and/or calcium concentrations [66]. Chicken annexin A5 shows a high sequence and structural similarity with mammalian annexins absent in chicken, such as annexins A3 and A4. Thus, some of the physiological functions exerted by these proteins may be carried out by chicken annexin A5, even those that could require calcium-dependent membrane aggregation.

Vesicle aggregation is negatively regulated by phosphorylation of the *N*-terminal domain at serine/threonine and tyrosine residues [13,20,67,68] or by proteolysis of the *N*-terminal extension [69]. In addition, the aggregation process may finally lead to fusion processes as described for annexin A7 regarding its involvement in exocytosis of lung surfactant, catecholamines, and insulin [70]. Phospholipid composition and calcium sensitivity for this aggregation activity significantly varies among individual annexins [2,38,65]. Moreover, the lipid composition and organization regulates the Ca^2+^-sensitivity of certain annexins for membrane bridging by modulation of membrane fluidity [71].

**Figure 3:**
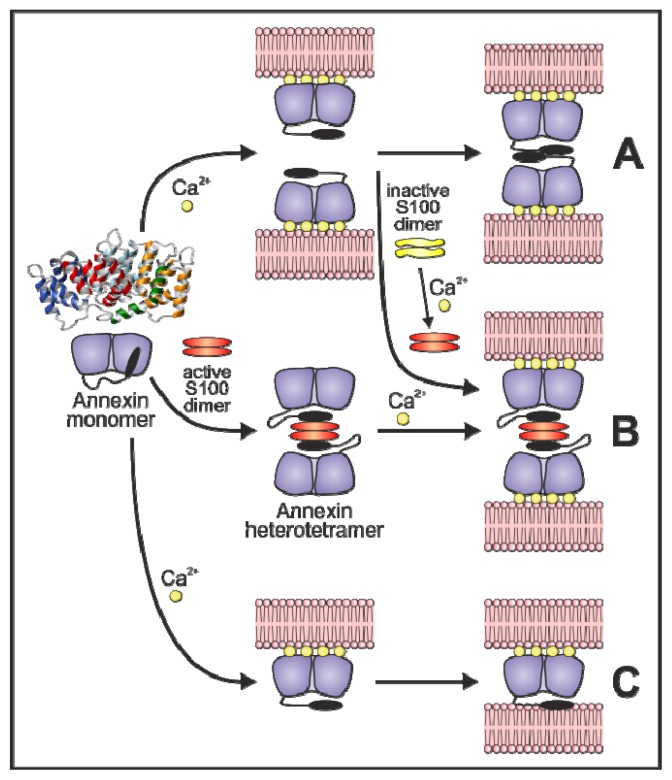
Proposed mechanisms for annexin-mediated membrane-vesicle aggregation. Several mechanisms have been proposed to explain the annexin-mediated induction of vesicle aggregation. Essentially, the mechanism depends on the molecular characteristics of the annexin involved in the process. In all cases, calcium seems to be essential to induce the process. (**A**) Aggregation induced by annexin-annexin interactions through the concave side of the molecules (probable involvement of the *N*-terminus); (**B**) Aggregation induced by the formation of heterotetrameric annexin bridges. Annexin A2 does not require calcium to interact with S100A10 as this small S100 molecule is permanently in the “activated” form without binding calcium. Other annexins require calcium for the formation of the complex with S100 proteins that are found in an “inactivated” form at low calcium concentrations (*i.e*., annexin A1 with S100A11); (**C**) Vesicle aggregation induced by the appearance of a second phospholipid- binding site in the *N*-terminus after calcium-dependent binding to one membrane and consequent annexin structural rearrangement. (Part of the figure is based on the model proposed in [1] for annexin A1).

How do annexins induce membrane aggregation? Several mechanisms have been proposed and are depicted in Figure 3: (A) self-association/dimerization of annexins bound to separate membranes; (B) establishment of heterotetramers formed by two annexins and two S100 proteins; or (C), the appearance of a second phospholipid-binding site after the interaction of specific annexins with membranes via the calcium-dependent type II sites located on the convex surface of these annexins.

Cryo-electron microscopy of aggregated lipid vesicles in the presence of wild-type annexin A4 shows a separation compatible with two layers of membrane-bound annexin A4 [20], in accordance with the first mechanisms described for vesicle aggregation. We have also demonstrated that chicken annexin A5 induces vesicle aggregation via establishment of protein dimers between molecules on different vesicles through the concave surfaces based on the kinetic analysis of the aggregation process together with cross-linking experiments at low-membrane occupancy and inhibition of vesicle aggregation by blockage of the concave annexin surface with heparin tetrasaccharide. Moreover, the essential role of the *N*-terminus of this protein for aggregation was put forward using chimeras with human annexin A5 (that does not aggregate vesicles) and truncation mutants [66].

Annexins A1 and A2 seem to be able to induce membrane aggregation via different mechanisms in which the *N*-terminal domain also plays an important role: (i) membrane bridging through heterotetramers (or octamers) with proteins of the S100 family (S100A11 and S100A10) [72,73], (ii) formation of dimers via interaction through the *N*-terminal domains of monomeric annexins, as suggested for annexin A4 and chicken annexin A5 [74], or (iii) interaction of one molecule with two adjacent membranes simultaneously [24,75,76]. Annexin A2 is more prone to form heterotetramers due to a constitutive dimeric active conformation of S100A10 under physiological conditions. However, at mildly acidic pH, this protein does not undergo the proposed structural rearrangement described for annexin B12 but experiments conformational changes that allow interaction with membranes in a calcium-independent manner. This leads to vesicle aggregation via exposure of a secondary hydrophobic binding site that allows membrane bridging as a monomer [76]. On the other hand, annexin A1 interacts with S100A11; this protein requires calcium binding to adopt an active conformation to form heterotetramers with annexin A1. The crystal structures of annexin A1 in the absence [22] and presence of calcium [24] (Figure 2) suggest a calcium dependent relocation of the *N*-terminal helices. Upon calcium binding, the *N*-terminal domain is exposed acquiring a very flexible conformation, as confirmed by molecular dynamics [77]. In the proximity of phospholipid membranes, the *N*-terminal domain refolds into α-helical structures that allow favorable direct interactions with the membrane providing a peripheral membrane anchor for vesicle aggregation [75].

Additional membrane aggregation mechanisms have been suggested for annexin A6. As we have previously commented, this protein is unique in the annexin superfamily as it consists of two annexin core modules connected by a 40 amino acid loop. Crystallographic data reveal that the two annexin cores are tilted with respect to each other in such a way that none of the two cores can bind to a membrane with its convex surface parallel to the membrane [78]. However, phosphorylation of a threonine residue in the connecting loop seems to increase the flexibility of the molecule, as deduced from the crystal structure and biochemical analysis of the phosphorylation-mimicking mutant T356D [79]. In fact, electron microscopic studies of membrane-bound annexin A6 2D crystals have determined that the two annexin cores reorient and become coplanar upon membrane binding [80,81]. More recently, and based on quartz crystal microbalance with dissipation monitoring, cryo-electron microscopy and atomic force microscopy techniques, a new model for the association of this annexin with membranes has been proposed. Authors suggest that at low calcium concentration (<150 μM), annexin A6 binds to membranes via the two coplanar annexin cores and cannot induce vesicle aggregation. However, at higher calcium concentration (~2 mM), a conformational switch allows annexin A6 to bind two adjacent phospholipid membranes due to the appearance of a dormant phospholipid binding sequence in the interconnecting loop, adopting a conformation similar to that observed in the crystal structures [82].

## 4. Functional Implications of Phospholipid Binding

**Figure 4:**
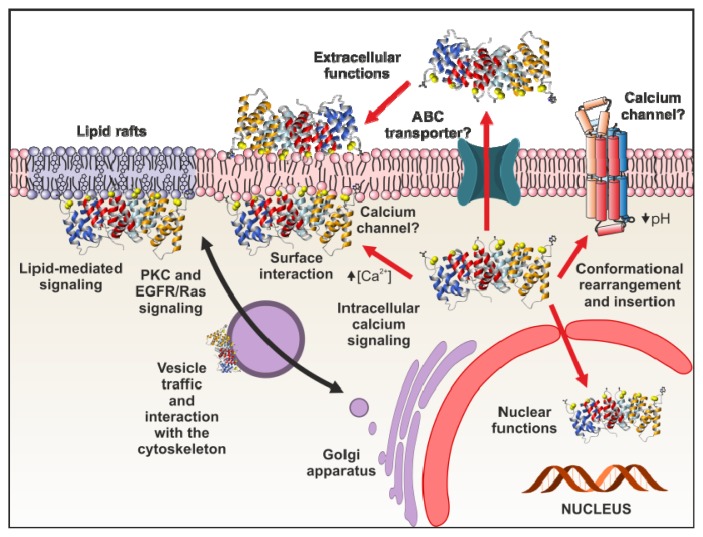
Intra- and extracellular functions of annexins. Annexins can be found in different intracellular compartments, including the nucleus, in equilibrium between soluble and a membrane-bound or cytoskeleton-bound forms depending on local intracellular calcium variations and lipid composition. In addition, acidification in the close proximity of the membrane due to acidic phospholipids can induce a conformational rearrangement of specific annexins with insertion of the annexin helices into the membrane. Although they lack signal sequences for secretion, they can be localized on the outer plasma membrane or soluble as circulating proteins where they can interact with biological membranes that expose PS, such as activated platelets.

Annexins have been proposed as membrane-membrane or membrane-cytoskeleton linkers, being implicated in Ca^2+^-regulated exocytosis events, endocytosis, cell signaling and stabilization of specific domains of organelle membranes and the plasma membrane. Moreover, annexins are thought to act themselves as Ca^2+^ channels although it is yet not clear how calcium permeability is achieved. However, other potential functions have been suggested taking into account that some of these proteins can migrate to the nucleus or be secreted to the extracellular space, acting as important regulators of several physiological processes, such as coagulation or inflammation. The cellular mechanism by which some annexins are secreted remains controversial. Annexins have no hydrophobic signal peptide and it has been suggested that annexin A1 may be secreted via ATP-binding-cassette (ABC) transporters [83,84]. On the other hand, annexin A5 circulating in the plasma may arise from damage of endothelial cells or trophoblasts [85]. Here we will briefly summarize different functions which are closely related to the ability of annexins to interact directly or indirectly with phospholipid membranes within the cell or at the extracellular space (Figure 4).

### 4.1. Intracellular Functions

#### 4.1.1. Annexin Interactions with the Cytoskeleton

Several members of the annexin superfamily have been described to interact with the cytoskeleton, mainly with actin. Annexin A2 was the first annexin shown to bind to actin filaments in a Ca^2+^-dependent manner. Moreover, this annexin possesses the ability to bundle F-actin filaments and plays an important role in their polymerization [86,87]. Another annexin that binds to F-actin is annexin A1, which also interacts with profilin, a G-actin binding protein and regulator of actin polymerization [88–90]. Interaction of annexin A5 with actin has also been reported in platelets, where it represents a key regulator of the coagulation process [91]. Finally, interaction of annexin A6 with actin has been described to be required for the dynamic reorganization of the sarcolemma during Ca^2+^-controlled smooth muscle contraction [92]. Regarding microtubules, a colocalization of annexin A11 with α-tubulin during mitosis has been described in COS-7 cells [93]. Migration of annexin A11 towards the nucleus is calcium-dependent and probably requires its interaction with calcyclin (S100A6). Once in the nucleus, annexin A11 plays an essential function for midbody formation and completion of the terminal phase of cytokinesis [94].

#### 4.1.2. Annexins as Membrane Scaffolds

It is well known that annexins are not only able to interact with membranes and cytoskeleton, but also to form lateral assemblies leading to the formation of two-dimensional crystal structures on the bilayers. In this regard, annexin A5 has the ability to bind in a calcium-dependent manner to PS membranes and to form a two-dimensional crystal lattice through the lateral interaction of protein trimers. It has been proposed that these structures may be involved in stabilizing certain plasma-membrane structures and in changing membrane curvature and cell shape. It is also well established that this type of membrane coating plays an essential role in the anticoagulant role of annexin A5 [95]. On the other hand, annexins A1 and A2 seem to associate into a different type of assembly when bound to PS:PC bilayers. It has been observed that the establishment of these annexin assemblies is accompanied by the segregation of membrane lipids, with certain negatively charged phospholipids accumulating underneath the annexin clusters, and that these annexins may even adsorb irreversibly to PS-enriched microdomains at high-calcium concentrations [96]. This activity could be responsible for an annexin-mediated formation of certain phospholipid domains in cells [38].

#### 4.1.3. Annexins in Vesicle Traffic

The key role of annexins in vesicle traffic is well known since the discovery and isolation of annexin A7 (synexin) that resulted to be essential in Ca^2+^-regulated chromaffin granule exocytosis [97]. Several additional annexins, including A1, A2, A3, A6, A7, A11, A13, and B7, have been linked to exocytotic processes, more specifically to post-trans-Golgi network events in the biosynthetic pathway [1]. For instance, annexin A2 has been proposed to promote the formation of lipid microdomains, which are essential for Ca^2+^-regulated exocytosis [98]. Regarding the endocytic pathway, some of the best-characterized annexins involved in this process are annexins A1, A2 and A6 [99]. Annexin A2 has been identified as an important element in the formation of early endosomes [87] and annexin A1 seems to participate in the internalization of multivesicular endosomes and epidermal growth factor receptor (EGFR) [100]. Besides annexin A1, other annexins are also involved in different steps that control the endocytic transport and signaling of the EGF receptor, such as annexins A2, A6 and A8 [101]. Annexin A6 is also involved in the regulation of the transport of cholesterol and, indirectly, the export of caveolin from the Golgi [102–104]. Finally, annexins A1 and A2 are considered important in phagocytosis, although other annexins, such as A6, A7 and A11, seem to be also involved in this process [105].

#### 4.1.4. Annexins and Intracellular Signaling

Annexins act as intracellular sensors discriminating incoming signals contributing in this way to the response of eukaryotic cells to the changing environment. Annexins interact with membranes at specific microdomains that are characterized by their lipid composition and structure [106,107]. As previously discussed, annexins preferentially associate with PS, but they also interact with other phospholipids, such as PA, PI, PIP_2_, PE, fatty acids (*i.e*., arachidonic acid), ceramides and lipid-derived metabolites [108]. Most of these molecules participate in lipid-mediated signaling pathways that regulate key cellular processes [109]. Thus, PIP_2_ is a major phosphoinositide of the plasma membrane that comprises about 1% of the plasma membrane phospholipids. Annexin A2 binds with high affinity to PIP_2_, which is often found in cholesterol-rich membrane domains, providing a link with actin cytoskeleton [40,41,99]. Since PIP_2_ is the precursor of the second messengers IP3 and diacylglycerol, annexin A2 could be involved not only in the regulation of membrane-cytoskeleton dynamics but also in other cell signaling events [110].

Acid sphingomyelinase converts sphingomyelin to ceramide under certain conditions in the presence of sustained elevated calcium concentrations. Upon its genesis, ceramide self-associates into platforms, promoting gross reorganization of the plasma membrane structure involving clustering of signaling molecules and an amplification of vesicle formation, fusion and trafficking. Thus, ceramide is considered a key lipid mediator in cellular processes such as differentiation, proliferation, growth arrest, and apoptosis [111]. Annexin A1 has been reported to be involved in the calcium-dependent production of ceramide and promotes its clustering into the membrane platforms. These effects are restricted to this annexin as they depend on its specific *N*-terminal domain [112].

Protein kinase C-α (PKCα) phosphorylates annexins A1, A2 and A4 modifying their calcium- and phospholipid-binding properties. Although annexin A6 interacts directly with PKCα, it is not phosphorylated. This annexin acts as a new scaffold for recruiting PKCα to the plasma membrane, which promotes PKCα/EGFR complex formation with consequent downregulation of ligand-induced EGFR signaling [110,113]. Annexin A5 has also been described as a negative regulator of PKC activity; this inhibition is not due to a direct interaction but is a consequence of phospholipid sequestration [114].

Several annexins have been reported to modulate the EGFR/Ras signaling pathway through different mechanisms. For example, annexin A6 inactivates Ras by recruiting p120GAP (GTPase activating protein) to the plasma membrane of breast cancer cells [115]. Other annexins involved in the regulation of the EGFR/Ras signaling and trafficking are annexins A1, A2 and A8. Annexins A1 and A2 are phosphorylation substrates of EGFR. In addition, these annexins may interact with several proteins involved in this and other signaling pathways [101,116,117].

#### 4.1.5. Annexins and Membrane Repair

Cell membrane repair is an active process that requires extracellular calcium, intracellular vesicles and calcium-dependent exocytosis. Besides calcium and cytoplasmic vesicles, several families of proteins (soluble NSF attachment protein receptors (SNAREs), synaptotagmins, ferlins and annexins) have been identified as part of the repair/resealing machinery [118]. For example, dysferlin, MG53, AHNAK, caveolin-3 and calpain-3 have been described to participate in skeletal muscle repair. The relevant role of annexins in plasma membrane repair and protection against membrane damage is beginning to be unraveled [119]. The first annexins known to be involved in this process were annexins A1 and A2, which interact with dysferlin [120,121]. More recently, it has been reported that the self-assembly of annexin A5 into two-dimensional arrays on membranes upon calcium activation promotes cell membrane repair [122]. In fact, the antiinflammatory, profibrinolytic and anti-thrombotic activities of these three annexins (A1, A2 and A5) have been related to the repair machinery that protects cells against damage derived from membrane rupture [123–125]. It has also been described that annexins may participate in the repair process with other activities, such as facilitating the transport of cytoplasmic vesicles along actin filaments or in the step of vesicle aggregation preceding fusion events. Annexin A6 is also involved in membrane repair forming part of a scaffold linking membrane microdomains with the cytoskeleton [126] and providing membrane permeability protection against chemical and physical stresses [119]. In summary, plasma membrane repair requires the formation of a complex array of protein-protein interactions as that described for the three-dimensional multiprotein complex that includes S100A10, annexin A2, and AHNAK, which along with dysferlin, functions in muscle and cardiac tissue repair [127].

#### 4.1.6. Annexins as Calcium Channels and Ion Channel Regulators

**Figure 5:**
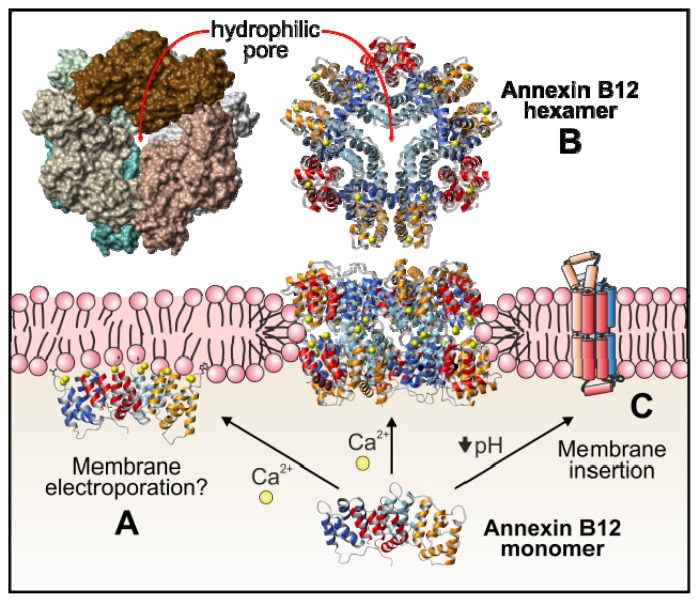
Proposed interactions of annexin B12 with cell membranes. Annexin B12 is quite similar to annexin A5 and it can interact with cell membranes in a superficial manner in response to an increase in calcium concentration. This interaction may induce alterations in the membrane and allow electroporation of calcium ions (**A**). It has also been proposed that a hexamer of annexin B12 (PDB file 1AEI; [129]) may integrate into the membrane in the presence of calcium (**B**), and could function as a calcium channel due to the existence of a central hydrophilic pore in the hexamer (ribbon and surface representations are shown from an upper view showing the hydrophilic pore). At low calcium concentration but in the presence of mild acidic pH in the proximity of the membrane, annexin B12 may experiment an overall structural rearrangement with formation of seven transmembrane helices that may allow the calcium channel activity (the helix distribution is based on a scheme in [1] and the work of Langen and coworkers [56–60]) (**C**).

Although the precise mechanism remains unknown, annexins exhibit calcium channel activity in plasma membranes and in matrix vesicles. Annexin A7 was the first one related to this function, followed by annexin A5, although it has been suggested that all annexins could act as calcium channels due to their central hydrophilic hole (Figure 1B), that is an structural feature common to all the members of this family of proteins [1,38]. However, *in vivo* data are scarce and it is complicated to explain how annexins can induce calcium permeability mainly taking into account the peripheral interaction of these proteins with membranes and the dimensions of the annexin monomers, which cannot expand the bilayer. It has been proposed that annexin monomers may destabilize the phospholipid bilayer inducing electroporation of the membranes and thus promoting ion permeability (Figure 5A) [128]. The analysis of *Hydra* annexin B12 has suggested two additional mechanisms. Initially, and based on the crystal structure of a hexamer of this annexin in the presence of calcium, the potential insertion of the hydrophilic hexamer into phospholipid bilayers was proposed. This insertion could induce a local reorientation of the bilayer phospholipids allowing a transmembrane structure that may be responsible for the calcium channel activity (Figure 5B) [129]. Later on, as previously discussed, the same group suggested the insertion of annexin B12 at mild acidic pH after undergoing a considerable conformational change. The hypothetical membrane-inserted annexin would have seven transmembrane domains and would therefore adopt the topology of a more conventional channel (Figure 5C) [56–60].

The role of annexins in the regulation of ion channels is less controversial than their activity as calcium channels. There is ample experimental evidence that annexins A2, A4 and A6 are modulators of plasma-membrane chloride channels and sarcoplasmic reticulum Ca^2+^-release channels [1,38]. Additionally, annexin A2 complexes with S100A10 are involved in the regulation of several other ion channels, as a neuron-specific Na^+^ channel, the TASK-1 K^+^ channel, or the epithelial Ca^2+^ channels TRPV5 and TRPV6. The complex S100A10-annexin A2 seems to be required for the trafficking of these ion channels from their intracellular sites to the plasma membrane [38].

### 4.2. Extracellular Annexin Activities

#### 4.2.1. Interaction with Virus and Extracellular Matrix Components

In a similar way to their ability to interact with the cytoskeleton within the cells, annexins are also able to bind extracellular elements thus affecting important cell functions and regulating different processes. One example is their ability to interact with viruses with the subsequent involvement in the infection process. The first annexin found to be involved in virus docking was annexin A5, which specifically interacts with the small hepatitis B virus envelope protein [130] and with influenza viruses [131]. Later on, annexin A2 was described to play an essential role in the production of infectious hepatitis C virus particles not only affecting their assembly [132], but also the formation of their replication complex [133]. Moreover, this annexin has also been shown to interact with the capsid protein VP1 of enterovirus 71, enhancing viral infectivity [134], and with HIV-1 Gag protein at the phosphatidylinositol 4,5-bisphosphate-containing lipid raft membrane domains at which Gag mediates viral assembly, favoring HIV-1 virus replication and production [135]. Annexin A5 has been identified in the exosomal compartment of HIV-1-infected and -uninfected lymphocytic H9 cells showing differential protein expression patterns. In addition, this annexin is associated with HIV-1 p24 and Tat proteins [136]. Interestingly, it has also been demonstrated that annexin A2-mediated enhancement of cytomegalovirus infection opposes inhibition by annexin A1 or annexin A5 [137]. The latter are not the only annexins described to inhibit virus infection, as human annexin A6 has also been demonstrated to interact with influenza A virus protein M2, negatively modulating infection [138].

On the other hand, annexins can also interact with other extracellular targets, including different extracellular matrix (ECM) elements. Thus, annexin A5 was described to interact with Type II and X collagens [139] and it was demonstrated that chondrocytes attach to Type II collagen using this annexin [140]. It was further revealed that this interaction is essential to regulate cartilage mineralization due to the calcium channel activity of annexin A5 (enhanced after interaction with collagen) present in matrix vesicles secreted by hypertrophic chondrocytes [141]. Cell surface annexin A2 also interacts with ECM components, as it was described as a high affinity receptor for the alternatively spliced segment of tenascin-C [142]. Another example is annexin A6. This protein contributes to the invasiveness of breast carcinoma cells by influencing the organization and localization of functional focal adhesions affecting the location of cadherins [143].

#### 4.2.2. Annexins and Inflammation

One of the best-characterized biological processes regulated by annexins is inflammation. Annexin A1 is able to inhibit different enzymes involved in the inflammatory process, including phospholipase A_2_ (PLA_2_), cyclooxygenase-2 (COX-2) and inducible nitric oxide synthase (iNOS). Moreover, once secreted, annexin A1 (or its cleaved *N*-terminal domain) binds to the formyl peptide receptor (FPR or ALXR), promoting cell detachment and inhibition of leukocytes migration, with the subsequent reduction in the inflammatory response. In addition to affecting the migration of leukocytes through FPR activation, extracellular annexin A1 is involved in apoptosis. It can trigger pro-apoptotic responses in neutrophils, and can function as an engulfment ligand that is presented on the surface when cells become apoptotic [144]. Moreover, cell-surface annexin A1 seems to be required for the clearance of the apoptotic cells, which can be mediated by PS receptors on the engulfing cells that possibly recognize annexin A1 or annexin A1-PS complexes [145]. Annexin A5 has also been described to inhibit PLA_2_ [146] and, taking into account its ability to interact with PS, it is able to inhibit the phagocytosis of apoptotic bodies by macrophages [147]. Annexin A6 also inhibits the cytoplasmic activity of PLA_2_. This activity is involved in Golgi vesiculation events and its inhibition in cells overexpressing annexin A6 interferes with caveolin export from Golgi membranes [148].

#### 4.2.3. Annexins in Coagulation and Fibrinolysis

An anticoagulant role has been proposed for extracellular annexin A5 based on its ability to form 2D crystalline shields on the surface of activated platelets that express PS on the outer leaflet of their membrane. This shield effectively sequesters PS from procoagulant factors that use this phospholipid in the clotting cascade [38]. In this regard, antibody-mediated inhibition of the anticoagulant property of annexin A5 has been proposed to occur in recurrent pregnancy losses observed in patients with lupus erythematosus and antiphospholipid syndrome [149]. Moreover, annexin A5 effectively unmasks the procoagulant surface of the placental syncytiotrophoblast to create a prothrombotic microenvironment. Thus, in patients with antiphospholipid syndrome it has been observed that the presence of anti-annexin A5 antibodies promotes placental thrombosis [150].

Annexin A2 plays an important role in fibrinolysis. It has been described that extracellular annexin A2 also forms heterotetramers with S100A10 on the cell surface. This cell surface complex acts as a co-receptor for plasminogen and tissue plasminogen activator (tPA), promoting the production of plasmin with the subsequent degradation of fibrin [151,152].

## 5. Annexin A5: A Tool in Research and Diagnostic

Labeled annexin A5 is currently used for apoptosis detection in cell cultures, and also for *in vivo* molecular imaging. The plasma membrane of living cells is a highly organized asymmetric three-dimensional system. The electrically neutral PC, sphingomyelin (SM) and glycosphingolipids are mainly located on the outer leaflet of the plasma membrane, whereas aminophospholipids such as PE and anionic PS and PA are exclusively present in the cytoplasmic or inner leaflet [153,154]. Local or global changes in lipid asymmetry are critical for several cellular events like membrane biogenesis, cell cycle progression, apoptosis, platelet coagulation and injury. The asymmetrical phospholipid location yields a strong distribution of electrostatic charge between the two membrane surfaces. The movement of phospholipid molecules between the two leaflets is maintained by the action of specific lipid-translocating proteins [153,155]. Thus, the membrane ATP-dependent flippases and floppases facilitate the translocation of lipid molecules from one leaflet to the other against their concentration gradient. In addition, the lipid exchange can be mediated by energy-independent scramblases being coupled to the translocation of calcium ions. Scramblases translocate the phospholipids bidirectionally over the two leaflets thereby collapsing PS asymmetry.

Apoptosis is a programmed type of cell death involved in a wide variety of physiological and pathological processes. The mechanisms involved in apoptosis have been extensively described in detail elsewhere [156,157]. Apoptosis can be triggered by different stimuli leading to loss of plasma membrane asymmetry, release of several mitochondrial factors, activation of cytoplasmic caspases, loss of mitochondrial membrane potential, condensation of chromatin, internucleosomal degradation of DNA and cell shrinkage with subsequent membrane blebbing. All these processes occur with the maintenance of cell integrity; when the cell is disintegrated, its cytoplasm is not released but is maintained in apoptotic bodies. The recognition and engulfment of the apoptotic cells by phagocytes involves “find-me” signals released by apoptotic cells and the exposure of “eat-me” signals that allow the clearance of such death cells [158,159].

The loss of phospholipid asymmetry in the plasma membrane, due to a redistribution of anionic PS from the inner membrane leaflet to the outer one, is one of the most remarkable features occurring during early apoptosis without compromising the barrier function of the cell membrane [160]. Different methods have been developed to detect the early PS expression on the extracellular face of the plasma membrane of apoptotic cells. This phospholipid can be detected by dye-labeled PS-binding proteins. One of the most popular noninvasive tools for the detection of apoptosis is the use of annexin A5 (commercially known as annexin V) labeled with a fluorescent dye [161–164]. The efficiency of annexin A5 as apoptosis marker is due to: (i) its high affinity for PS (nanomolar range) in the presence of calcium, (ii) the successful expression in bacterial systems of the recombinant protein which allowed the generation of commercially available assay kits [165], and (iii) the description of methods of conjugation of this protein to fluorescein isothiocyanate (FITC) and similar fluorophores [166]. Furthermore, this assay is able to discern between apoptotic cells and necrotic cells, which have compromised plasma membrane integrity, after propidium-iodide DNA staining. In this way, viable, apoptotic, and necrotic cells can be discriminated by either fluorescence microscopy or flow cytometry.

Molecular imaging of both apoptosis and necrosis is useful to understand the cell death process in several pathologies such as acute myocardial infarction, cerebral stroke and atherosclerosis, as well as for the measurement of drug response in cancer patients. The detection of PS is the biological property by which the development of several radiolabeled derivatives of annexin A5 has been described as a diagnostic tool. Thus, radioisotopes (*i.e.*, technetium and halogens), their conjugation methods, and quality control of the radiopharmaceuticals have been described [161,167,168]. In fact, radionuclide labeling of annexin A5 has allowed to image apoptosis *in vivo* and therefore its use as a tool in diagnostic techniques, treatment evaluation, and therapy approaches monitoring cell death-inducing or cell death-preventing therapies [161,169].

Recently, annexins (*i.e*., A1, A2, A5 or A6) have been reported to be present in exosomes from different cellular origins being mainly involved in exosomal uptake or recycling mechanisms [136,170–172]. Independently of the roles of these proteins in exosomes, annexin A5 has been used as a research tool to demonstrate the requirement of PS exposure for exosome uptake. For example, blockage of PS from neuron-derived exosomes (which are associated with extracellular amyloid β-peptide) using this annexin not only prevented exosome uptake but also suppressed amyloid β-peptide incorporation into microglia [173].

## 6. Conclusions

Annexins comprise a multigene superfamily of calcium-regulated membrane binding proteins expressed widely throughout the animal and plant kingdoms. Humans express twelve different annexins, some of them with splicing variants, which exert differential functions. But all of them contain a conserved homologous calcium-membrane binding core that allows the peripheral docking of these proteins to membranes. This docking can occur at high density with establishment of two-dimensional arrays of annexins that condition membranes by formation of phospholipid microdomains with specific chemical and structural properties. On the other hand, when interacting at lower density, they can modulate membrane permeability or trigger or regulate important intracellular signaling events. In addition, annexins may be secreted or released from damaged cells to exert important extracellular functions as the regulation of inflammatory reactions, coagulation or the fibrinolytic homeostasis. In any case, although the major common feature of annexins is their homologous protein core, the variable *N*-terminal extension plays key roles in the regulation of the specific functions of individual annexins or may even be responsible of additional features of some of these proteins.

The existence of this wide variety of homologous annexins in one organism allows the redundancy of cellular functions by some members of this superfamily of proteins, as revealed by knockout experiments. However, specific annexins respond to a large variety of different stimuli, mainly related to changes in calcium concentration, allowing a fine tuning of each annexin to respond only under certain circumstances. Although a lot is known regarding the *in vitro* activities of annexins mainly related to calcium-driven membrane interactions, *in vivo* functions are just beginning to be understood thanks to experiments using live cell imaging and targeted gene disruption in cells or in mice. Thus, roles have now been unequivocally established for annexin A1 in inflammation, annexin A2 in vesicle traffic and annexin A7 in regulation of cell growth. Additional important physiological functions may still arise as this family of proteins has been highly conserved during evolution and most organisms contain multiple members of the family that may act as potential functional backups. However, there is still a long way to go to understand the precise functions of individual annexins and it may prove a difficult job taking into account the potential redundancy of annexin functions, their involvement in multicomponent membrane-associated scaffolds and their interaction with several signaling pathways.
